# The mitochondrial genome of the gold-ringed cowry *Monetaria annulus* (Mollusca: Gastropoda: Cypraeidae) determined by whole-genome sequencing

**DOI:** 10.1080/23802359.2019.1627946

**Published:** 2019-07-11

**Authors:** Hiroaki Fukumori, Hajime Itoh, Takahiro Irie

**Affiliations:** a Sesoko Station, Tropical Biosphere Research Center (TBRC), University of the Ryukyus, Motobu, Okinawa, Japan;; b Atmosphere and Ocean Research Institute (AORI), The University of Tokyo, Kashiwa, Chiba, Japan;; c National Institute for Environmental Studies, Tsukuba, Ibaraki, Japan

**Keywords:** Caenogastropoda, Cypraeoidea, Littorinimorpha, next-generation sequencing, phylogenetic tree

## Abstract

The mitochondrial genome of the cowry snail *Monetaria annulus* (Caenogastropoda: Cypraeoidea: Cypraeidae) was determined by whole-genome next-generation sequencing. The mitogenome is composed of 13 protein-coding genes (PCGs), 2 ribosomal RNA (12S and 16S) genes, and 22 transfer RNA genes (tRNAs). This gene order is consistent with the previously published mitochondrial genomes of other species belonging to the order Littorinimorpha. The superfamily Cypraeoidea was recovered as a sister clade to the group of Tonnoidea and Neogastropoda.

Members of the marine gastropod family Cypraeidae (cowries) are commonly found on rocky shores and coral reefs in tropical and subtropical waters (Meyer 2003; Irie 2006). The superfamily Cypraeoidea comprises five families (Bouchet et al. 2017) and mitochondrial genome (mitogenome) has been sequenced only in a cypraeid species, *Erosaria spurca* (Osca et al. 2015; 11,107 bp, but approximately 3,000–5,000 bp are missing). The phylogenetic position of Cypraeidae within the superfamily is still controversial due to the scarcity of available molecular data (e.g. Ponder et al. 2008). The gold-ringed cowry *Monetaria annulus* (Linnaeus, 1758) is an intertidal species distributed throughout the tropical and subtropical Indo-West Pacific region (Irie and Morimoto 2016). In this study, we provide the first mitochondrial genome sequence of this species, including all protein-coding (PCG), transfer RNA (tRNA), and ribosomal RNA (rRNA) genes.

A single individual of *M. annulus* was collected from Sesoko on Okinawa Is., Japan (26°38′04″N, 127°51′51″E) in December 2016. The voucher specimen (IR002-Mannu) was deposited in Atmosphere and Ocean Research Institute, University of Tokyo. Genomic DNA was extracted from muscle tissue and was sequenced using a Hiseq X ten System (Illumina, San Diego, CA) after the preparation of the 10× Chromium genome library (10× Genomics, Pleasanton, CA) at Macrogen Japan Co. (Kyoto, Japan; order number: 1706AHX-0016). A total of 751,087,546 paired-end reads were assembled into 579,168 contigs by using Supernova v2.0.1 (Weisenfeld et al. 2017). Only one contig (16,086 bp; contig name: contig_323406) from the assembled data was identified as the mitochondrial DNA sequence, but tandem repeat sequences in the non-coding region was missing. The length of remaining gaps was confirmed by PCR-based techniques with Sanger sequencing and DNA fragment analysis with Capillary Electrophoresis. Consequently, at least 132 AT-repeats (> 264 bp) were found to be present in the non-coding region, although the exact length of the repeat sequences could not determined. The mitogenome sequence with all PCG, tRNA, and rRNA genes from the contig assembled by Supernova (contig_323406) was deposited in the DNA Data Bank of Japan (DDBJ) under the accession number LC469295.

This mtDNA sequence consists of 16,087 bp and contains 13 PCGs, 22 tRNAs, and 2 rRNA (12S and 16S) genes. Of these 37 genes identified, 8 tRNAs are encoded on the L-strand and the other genes are encoded on the H-strand. The 12S (975 bp) and 16S (1,407 bp) genes are located between tRNA^Glu^ and tRNA^Leu^. All PCGs contain ATG as the start codon, and TAA, TAG or TA– as the stop codon. Gene overlaps are observed between three pairs of genes, NAD5–tRNA^Phe^, CYTB–tRNA^Ser^, and NAD4L–NAD4, with the overlapped size of 1, 2, and 7 bp, respectively. The lengths of 22 tRNAs range from 66 to 71 bp. The gene order for *M. annulus* is the same as the previously reported mitogenomes of many other species belonging to the order Littorinimorpha (Osca et al. 2015). Figure 1 shows the phylogenetic position of *Monetaria annulus* within the subclass Caenogastropoda based on the present and previous mitogenome data.

**Figure 1.**
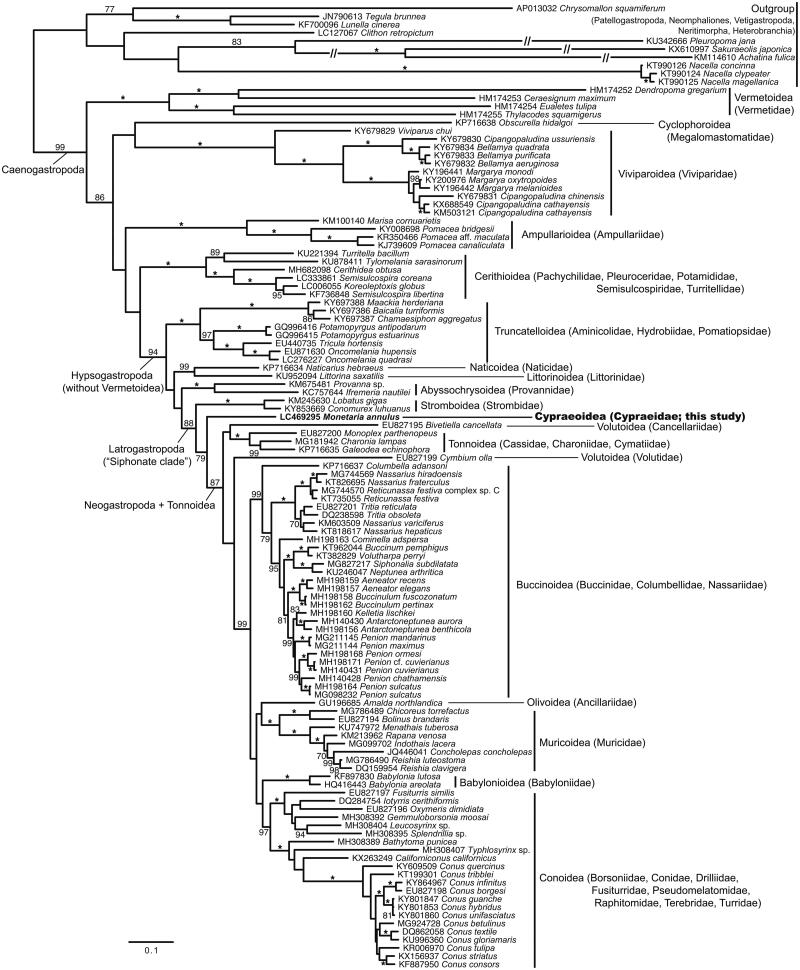
Maximum likelihood phylogeny of the subclass Caenogastropoda based on mitochondrial amino acid sequences of 13 protein-cording genes from *Monetaria annulus* (bold, this study; LC469295) and the 117 gastropod species previously published (one neomphaloid, two neritimorph, two vetigastropod, two heterobranch, three patellogastropod, and 107 caenogastropod species). Sequences were aligned separately for each gene using MUSCLE (Edgar 2004) in Translator X (Abascal et al. 2010) with default parameters. Ambiguously aligned positions were removed using Gblocks v.0.91b (Castresana 2000) with an option to allow gap positions in the final blocks for a less stringent selection. Tree reconstruction was performed in RAxML-HPC v.8.2.10 on XSEDE (Stamatakis 2014) using MTRev + G model through the CIPRES Science Gateway (Miller et al. 2010); nodal support estimated by 1,000 thorough bootstrap replicates. Scale bar represents branch length (substitutions/site). Accession numbers are shown in the tree. Asterisks indicate the node with 100% boot-strap probability (BP). BP values for each node were not shown below 70%. The higher classification follows Osca et al. (2015) and Bouchet et al. (2017). The superfamily Cypraeoidea was recovered as a sister group to the clade including Tonnoidea and Neogastropoda, although the bootstrap value was not high (BP = 79%). The superorder Latrogastropoda (Cypraeoidea + Stromboidea + Tonnoidea + Neogastropoda) was monophyletic (88%), as indicated by previous studies (Osca et al. 2015; Bouchet et al. 2017).
